# How different cilia beat frequencies impact on Kupffer's vesicle fluid flow

**DOI:** 10.1186/2046-2530-1-S1-P42

**Published:** 2012-11-16

**Authors:** R Rua, A Guerrero, SS Lopes

**Affiliations:** 1 Faculty of Medical Sciences, CEDOC, Portugal; 2 Instituto Gulbenkian Ciência, Portugal

## 

Motile cilia need to be coordinated and ciliary beat frequency (CBF) is characteristic of different types of cilia depending on their physiological function. In zebrafish, monociliated cells arise in the tailbud at the end of gastrulation in a transient spherical organ called Kupffer's vesicle (KV). Using zebrafish as a model, our group has been studying cilia length regulation and motility in wild-type (wt) and *aei-/-* mutant embryos. These mutants carry a premature stop codon in the deltaD gene. Recently, our group showed that Notch signaling was directly involved in the control of cilia length in the KV cells given that the *aei-/-* mutant present shorter cilia in KV cells. The goal of this project is the characterization of the CBF and beat patterns of *aei-/-* KV cilia vs. wt cilia. We did spectral analysis of individual cilia associated with high-speed digital videomicroscopy. By decomposing and comparing the obtained frequencies with Fourier Transform we have identified significant differences in KV cilia motility pattern between the wt and the *aei-/-* mutants. So far, we show that not only are the cilia shorter in the KV *of aei-/-* mutants but also their motility pattern is different resulting in an overall destructive fluid flow.

http://www.cedoc.org

